# The process of reaching psychological adjustment among adult women diagnosed with metastatic breast cancer and receiving cancer pharmacotherapy

**DOI:** 10.1016/j.apjon.2023.100184

**Published:** 2023-01-05

**Authors:** Chihiro Iseki

**Affiliations:** a Osaka Medical and Pharmaceutical University Graduate School of Nursing, Takatsuki-shi, Osaka, Japan; b Hyogo Prefectural Nishinomiya Hospital, Nishinomiya-shi, Hyogo, Japan

**Keywords:** Metastatic breast cancer, Cancer pharmacotherapy, Nursing, Psychological adjustment

## Abstract

**Objective:**

This study clarified the process by which adult women diagnosed with metastatic breast cancer (MBC) and undergoing cancer pharmacotherapy reach psychological adjustment.

**Methods:**

A semistructured interview was conducted with adult women who had received their MBC diagnosis. The data collected were analyzed using Kinoshita's modified grounded theory approach.

**Results:**

A total of 21 women with an average age of 50 years participated in the study. Seven categories and 21 concepts were generated through the analysis. Upon being diagnosed with MBC by a doctor, the participants felt the “threat of death” and “conflict with painful cancer pharmacotherapy.” Thereafter, they received “encouragement from strong supporters,” consolidated their “resolve to save their life,” and began cancer pharmacotherapy. During the therapy, they made “efforts to internalize MBC” to overcome the distress arising from the “struggle to internalize MBC,” and this led to the “expansion of self-awareness.”

**Conclusions:**

Despite finding themselves in harsh circumstances, the participants remained focused on the big picture and realized that cancer had changed their values and outlook on life, leading to psychological growth. It is important for nurses to provide systematic and continuous support from the time of MBC diagnosis.

## Introduction

Breast cancer is the leading cause of morbidity among women, not just in Japan but also worldwide.^1^ Approximately 500,000 women die from metastatic breast cancer (MBC) every year.^2^ With the development of new drugs in recent years, cancer pharmacotherapy has been successful in treating MBC and is expected to prolong patients’ lives. Therefore, patients with MBC must continue treatment for a long time until the treatment is successful.

Patients with MBC are conscious of death when diagnosed with MBC.^3^, ^4^, ^5^, ^6^ The incidence of anxiety and depression in patients with early breast cancer was 37.5% and 43.8%, whereas that of anxiety and depression in patients with recurrent breast cancer was 52.4% and 71.4%, respectively,^7^ indicating that patients with MBC have a higher incidence of anxiety and depression than patients with early breast cancer.^8^ In addition, patients with MBC suffer from the sequelae of the cancer pharmacotherapy they received for their initial treatment,^5^ and many of their physical problems have been reported to be associated with the onset of anxiety and depression.^9^ Furthermore, patients with MBC have been noted to have a low quality of life (QOL) due to the complex physical symptoms associated with metastasis and the added side effects of cancer drug therapy.^10^

The peak age of breast cancer in Japan is 45–54 years, and breast cancer is more common among women in adulthood.^11^ Since breast cancer metastasis tends to occur within 2 years of diagnosis of early breast cancer,^12^ it can be predicted that the number of patients with MBC during adulthood is high in Japan. In recent years, Japanese women have become more highly educated and effectively employed, resulting in later marriage and childbearing.^13^ As a result, women in adulthood are taking on gender roles such as child-rearing and caregiving at home, as well as important positions in the workplace. It has been shown that the prevalence of anxiety and depression is higher among women with breast cancer during adulthood than during old age with breast cancer.^8^^,^^9^ Thus, if adult women are diagnosed with MBC, they will have to deal with the complex physical issues associated with ongoing cancer pharmacotherapy, which will make role performance difficult and stressful.

Previous studies have identified the lived experiences,^3^, ^4^, ^5^, ^6^ prevalence of anxiety and depression,^7^^,^^9^ and emotional needs^14^ of patients with MBC. However, no empirical studies have focused on the process of reaching psychological adjustment among adult women diagnosed with MBC. Additionally, patients with MBC have often reported dissatisfaction due to the lack of professional support they received after their diagnosis.^15^ In order to provide professional support that improves the mental health and QOL of adult women with MBC, elucidating what phenomena occur during the process of psychological adjustment after the diagnosis of MBC is important.

Thus, this study clarified the process by which adult women diagnosed with MBC and undergoing cancer pharmacotherapy reach psychological adjustment.

## Definition of terms

In this study, psychological adjustment to MBC refers to the state of psychological stability achieved by coping with MBC and cancer pharmacotherapy. It also denotes self-integration and the alleviation of anxiety, depression, and negative emotions, which are stress reactions stemming from MBC and cancer pharmacotherapy.

## Methods

### Study design

This qualitative study used Kinoshita's modified grounded theory approach (M-GTA).^16^ M-GTA is a method that can set up an analytical focus person, generate explanatory concepts from data interpretation without data segmentation, and derive a theory within a limited range after examining the relevance of the generated concepts. M-GTA focuses on social interactions between humans and is suitable for areas where the phenomena to be studied have process characteristics. Therefore, M-GTA was considered suitable for this study.

### Population and sampling

The study targeted adults who were diagnosed with metastasis to distant organs after their initial treatment for breast cancer and were receiving cancer pharmacotherapy. The study included only (1) women aged 20–65 years who were diagnosed with MBC by a doctor, (2) those who had been diagnosed with MBC for more than 3 months but less than 3 years, (3) patients who were judged by the attending physician to be in a psychologically stable condition, and (4) those who were able to communicate verbally in Japanese. Patients with MBC with (1) a history of psychological illness, (2) severe anxiety or depression, or (3) severe physical symptoms were excluded from the study. Ultimately, 21 participants were included in the study.

### Instrument and data collection

The survey was conducted from May 2021 to June 2022. Interview surveys and the lifeline method^6^^,^^17^ were used for data collection. The lifeline method is a technique that can capture the dynamics of an individual's life journey, including their inner life.^17^ The vertical axis of the lifeline diagram represents the state of psychological adjustment, and the horizontal axis represents the passage of time since the diagnosis of MBC. Study participants were asked to draw a line through the diagram to indicate changes in their psychological state. A semistructured interview was conducted based on the interview guide, focusing on the psychological states indicated in the lifelines. During the interviews, the participants were asked to talk freely about how they felt when they were diagnosed with MBC, their treatment experience, and how their feelings changed over time. The interviews were conducted in a private room and recorded with the participants' permission. The participants were interviewed once or twice at most, and each interview lasted about 30–60 ​min.

In this study, to clarify the process from the time of diagnosis of MBC to psychological adjustment in adult women, it was considered important to objectively capture and analyze not only the participants' narratives but also their state of psychological adjustment. By comparing the content of their narratives with their state of psychological adjustment, the stage of the process the participants had reached was confirmed. It has been reported that psychological adjustment predicts general psychological distress such as anxiety/depression and individuals' level of QOL.^18^ Therefore, the Hospital Anxiety and Depression Scale (HADS)^19^ and the Functional Assessment of Cancer Therapy-Breast cancer module (FACT-B)^20^ were used as measures of psychological adjustment in this study. The HADS^21^ and the FACT-B^22^ have been translated into Japanese and have gained reliability and validity. The HADS consists of two factors, anxiety (seven items) and depression (seven items), for a total of 14 items; a score of 0–7 indicates “no anxiety or depression”; 8 to 10, “suspicious”; and 11 or more, “anxiety or depression.” The FACT-B consists of 36 items in five subscales (Physical well-being, Social well-being, Emotional well-being, Functional well-being, and Breast Cancer Scale). The total score (including the five subscales) ranges from 0 to 148, with higher values indicating better QOL. These scales were used after receiving approval from their respective developers. Information on the participants' age, site of metastasis, doctor's notes, and course of treatment was obtained from their respective medical records.

### Data analysis

The data obtained from the interview survey were analyzed using Kinoshita's M-GTA method.^16^ The analysis focused on adult women diagnosed with MBC after they completed their initial treatment. The theme of the analysis was the process from MBC diagnosis to decision-making for cancer pharmacotherapy and the process of reaching psychological adjustment through repeated treatment.

The analysis proceeded as follows: first, one participant who had a wealth of content related to the analysis theme was selected. The points of interest related to the analysis theme were extracted and a concept was generated. An analysis worksheet was created for each concept, which included the concept's name, definition, specific examples, and theoretical notes. From the second case onward, the similarities and differences between cases were examined. Next, categories and subcategories were created by examining the relationship between the generated concepts. Finally, a result diagram and storyline were created by considering the relationship between concepts and categories. The participants' HADS and FACT-B scores were also calculated.

### Ensuring authenticity of analysis

To ensure the veracity of the analysis, the author was supervised by researchers familiar with cancer nursing and qualitative research throughout the analytical process. When conducting the analysis, the data were repeatedly reviewed to examine the concepts and categories. Furthermore, to enhance the validity of the analysis, its trajectory from extracting specific examples (variations) from the data and generating concepts and categories to creating the storyline through analytical worksheets and theoretical memos was shown.

### Ethical considerations

This study was approved by the ethics review board of Osaka Medical and Pharmaceutical University (IRB No. 2924). In addition, the research was conducted with the approval of the ethical review board of each cooperating institution. The participants were informed of the study's purpose and methods, that their participation was voluntary, and that they could stop or withdraw at any time. Participants' confidentiality was ensured in this study.

## Results

### Demographic data

Table 1 presents an overview of the characteristics of 21 participants. The average age of the participants was 50 years. Twenty of them were living with their family and 13 were still employed. The most common site of metastasis was the lung, and about half of them had metastasis to multiple sites. Six patients were on chemotherapy and 15 were on endocrine and molecular targeted therapy. The average HADS-A, HADS-D, and FACT-B scores of the participants were 6.04, 5.48, and 99.6, respectively. Three participants had an anxiety score of 11 or higher on the HADS-A, and one participant had a depression score of 11 or higher on the HADS-D.

**Table 1:** Demographic data.

ID	Age, years	With family	Children	Site of metastasis	Treatment for early-stage breast cancer	Current drug treatment	HADS-A（0–21）	HADS-D（0–21）	FACT-B (0–148)
A	50	Yes	Yes	lung, liver, bone, brain	None	MT	5	6	89
B	50	Yes	Yes	bone	Chemo ​+ ​MT	MT	4	3	118
C	30	Yes	No	brain, bone	ET	Chemo ​+ ​ET	8	8	89
D	60	Yes	Yes	lung, liver, bone	Chemo ​+ ​ET	Chemo	7	4	128
E	50	Yes	Yes	bone	Chemo ​+ ​ET	ET ​+ ​MT	0	5	107
F	50	Yes	Yes	liver	ET	Chemo	8	5	99
G	50	Yes	Yes	lung	Chemo	MT	11	8	84
H	40	Yes	No	lung	Chemo ​+ ​ET	ET ​+ ​MT	4	3	100
I	50	Yes	No	lung	ET	ET ​+ ​MT	15	10	63
J	40	Yes	Yes	lung	Chemo ​+ ​ET	ET ​+ ​MT	5	7	94
K	40	Yes	Yes	bone	ET	ET ​+ ​MT	2	2	123
L	60	Yes	Yes	lung	Chemo ​+ ​ET	ET	4	1	130
M	30	Yes	Yes	lung	Chemo ​+ ​ET	ET ​+ ​MT	18	15	53
N	50	Yes	Yes	lung	ET	Chemo	8	8	73
O	40	Yes	Yes	lung	Chemo ​+ ​ET	ET ​+ ​MT	4	4	114
P	40	Yes	Yes	bone	ET	ET	5	5	105
Q	40	Yes	Yes	liver, bone	ET	ET ​+ ​MT	5	6	94
R	50	Yes	Yes	lung	Chemo	Chemo	3	5	89
S	60	Yes	Yes	liver, bone	Chemo ​+ ​ET	Chemo	5	4	92
T	50	No	Yes	lung, bone	Chemo	ET ​+ ​MT	4	2	129
U	40	Yes	Yes	lung	Chemo ​+ ​MT	ET ​+ ​MT	2	4	118

ET: Endocrine therapy, Chemo: Chemotherapy, MT: Molecularly-targeted therapy.

### The process of reaching psychological adjustment in patients with MBC

**Fig. 1:**
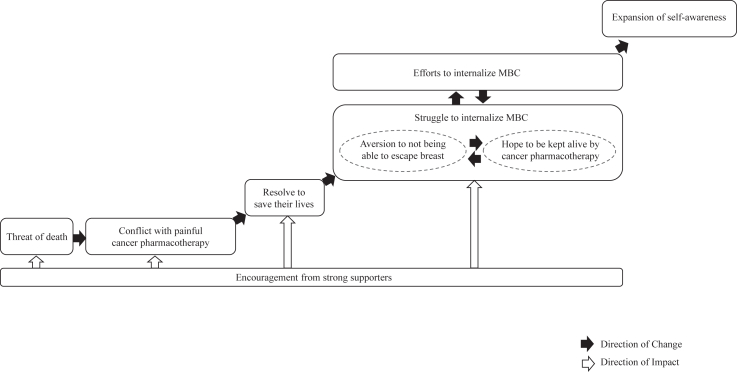
Psychological adjustment process of adult women diagnosed with metastatic breast cancer and receiving cancer pharmacotherapy.

The analysis generated 24 concepts, 2 subcategories, and 7 categories (Table 2). The process of reaching psychological adjustment for adult women diagnosed with MBC after completion of initial treatment is shown in Fig. 1. The following is a textual storyline of the relationships among the categories.

**Table 2:**
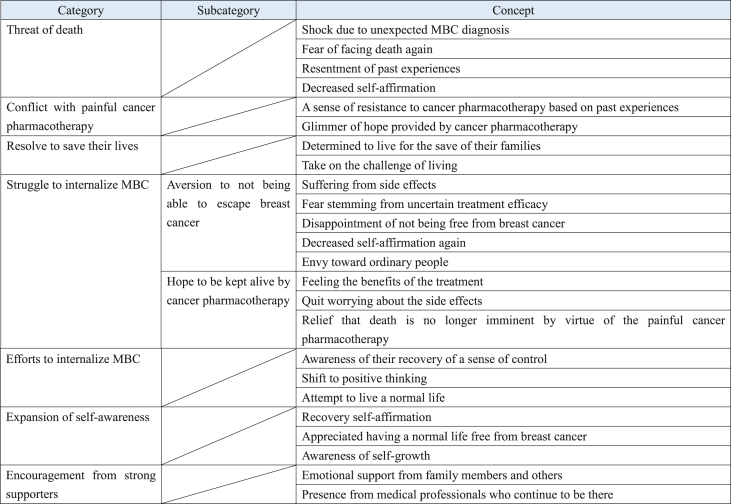
The process of reaching psychological adjustment in patients with metastatic breast cancer.

#### Storyline (Fig. 1)

When participants were diagnosed with MBC and informed about their course of treatment, they experienced a “shock due to unexpected MBC diagnosis,” “fear of facing death again,” “resentment of past experiences,” and “decreased self-affirmation.” In other words, they experienced the “threat of death.” Thereafter, while the participants had “a sense of resistance to cancer pharmacotherapy based on past experiences,” they were also in “conflict with painful cancer pharmacotherapy” because of the “glimmer of hope provided by cancer pharmacotherapy.” However, thanks to the “encouragement from strong supporters,” the participants were “determined to live for the save of their families” and decided to “take on the challenge of living.” In other words, the participants had the “resolve to save their lives” and began cancer pharmacotherapy.

Once the treatment commenced, the participants began “suffering from side effects” and experiencing “fear stemming from uncertain treatment efficacy,” resulting in “disappointment at not being free from breast cancer,” “decreased self-affirmation again,” and “envy toward ordinary people.” In other words, the participants experienced “aversion to not being able to escape breast cancer.” However, upon “feel the benefits of the treatment,” the participants “quit worrying about the side effects” and were “relief that death is no longer imminent by virtue of the painful cancer pharmacotherapy.” In other words, the participants began to embrace the “hope to be kept alive by cancer pharmacotherapy.” While continuing treatment, the participants alternated between “aversion to not being able to escape breast cancer” and “hope to be kept alive by cancer pharmacotherapy.” In other words, the participants experienced a “struggle to internalize MBC.” With the “encouragement from strong supporters” in these circumstances, the participants were able to gain “awareness of their recovery of a sense of control,” which allowed them to “shift to positive thinking” and made them “attempt to live a normal life.” In other words, the participants began “efforts to internalize MBC.” While alternating between the “struggle to internalize MBC” and “efforts to internalize MBC,” participants “recovery of self-affirmation,” “appreciated having a normal life free from breast cancer,” and gained “awareness of self-growth,” leading to an “expansion of self-awareness.”

Participants G, I, and M, who scored 11 or higher on the HADS with anxiety and depression, had not yet reached an “expansion of self-awareness,” while the other participants had done so.

#### Category features

(1) The threat of death

This category involved the sudden MBC diagnosis, facing the possibility of death that was presumed to be far away because of cancer pharmacotherapy preventing metastases, and being threatened with imminent death.

I don't even remember what I was thinking because I was so shocked. I was more shocked than when I was first diagnosed with breast cancer. I could not think of anything about the future. “Why did it metastasize to my lungs?” was the question in my mind. I was crying because I was so shocked. I was finally living a normal life, and it felt like everything was taken away from me (after being diagnosed with MBC) (Participant M).

I was very depressed. I talked with people who had no idea what had happened to me nine years ago and told them that I had already recovered. At first, I was stunned and did not understand what the condition meant. I could not accept it (Participant I).

(2) Conflict with painful cancer pharmacotherapy

This category involved the confusion between the resistance to cancer pharmacotherapy with painful side effects in the past and the possibility of survival by receiving the treatment.

The doctor explained that I needed to undergo cancer pharmacotherapy, so I realized that the same treatment would be given again and wondered if it would really work. (Omitted). I was a little desperate, and I wondered if I would have to go through the side effects again, such as my hair falling out. However, I thought that it was not the end and that I could receive the treatment (Participant B).

I felt like I was starting all over again. Chemotherapy was the worst. I was really anxious about losing my hair again. I was worried that it would be harder than the first time (Omitted). When I heard about it from the doctor, I felt uneasy because it was a new drug. However, since it was a new drug, I also thought that it might be effective (Participant H).

(3) Resolve to save their lives

This category involved accepting the side effects of painful cancer pharmacotherapy and preparing for the treatment in order to survive.

I'm resigned to this. I have no choice but to follow the doctor's advice and receive the treatment. I have no choice but to do that. Hesitation does not help the situation, nor does crying, because it would not cure the cancer. In that case, I thought that I had no choice but to receive the treatment and be positive (Participant U).

I felt like my only option was to undergo the treatment, and I'll just have to do my best, even more so because my children were crying too (Participant R).

(4) Struggle to internalize MBC

This category involved a strong rejection of the fact that the breast cancer has metastasized and the suffering from not being able to accept the deadly MBC as one's own problem. It comprised the subcategories “aversion to not being able to escape breast cancer” and “hope to be kept alive by cancer pharmacotherapy.” “Aversion to not being able to escape breast cancer” referred to experiencing strong negative feelings in the face of breast cancer. “Hope to be kept alive by cancer pharmacotherapy” referred to placing one's hope on life despite the painful cancer pharmacotherapy.

There are fluctuations in the urge to do my very best. If I get tired, I am less motivated to do my best, and if my body feels better, I am more motivated to work hard. There are these kinds of fluctuations. Although I try to defy the disease and commit to doing my best, I cannot maintain this sentiment. So, there are fluctuations (Participant B).

The tumor marker value did not seem to be going down. I thought I would die of cancer. It shocked me. The treatment didn't give me hope (Participant E).

I thought that in the end it would only go in the direction of death. I thought I would never go back (Omitted). Waking up in the morning, I'm annoyed that the reality hasn't changed after all. That's why I'm afraid of mornings. When I wake up in the morning, I wish everything would change. In the end, I was like, “Oh, it's cancer,” “I'm a person with cancer.” I feel envious of ordinary people (Participant M).

The first computed tomography (CT) scan after I started taking the medicine showed that the tumor had shrunk by about 30% overall. The medicine (its side effects) was tough, but I felt rewarded. I was very hopeful since (the tumor) had become smaller (Participant I).

(5) Efforts to internalize MBC

This category involved the refusal to deny MBC and attempt to live a normal life as much as possible while accepting the disease as a part of oneself.

Maybe that’s what I thought. I thought and thought. Because I got depressed down to the bottom. I’ve thought it all out, so I have nothing more to think. All I have to do is move forward (Omitted). You could say that I was able to face my illness, or that I had to face it; it changed to that kind of feeling (Participant B).

I hate having breast cancer, but I feel lucky to have breast cancer. Research is so advanced, there are so many drugs out there, and things change year by year. I think so myself and tell my children. I want to convince myself of what I said. Like saying to myself that it’s going to be okay. (Omitted) I'm trying to think that it can't be helped even if I think about it consciously (Participant O).

(6) Expansion of self-awareness

This category involved experiencing personal growth as one objectively gained the ability, perceived oneself as suffering from MBC, achieved new realizations, and positively changed one's consciousness.

I think my consciousness has changed. I feel like I just can't sit around and do nothing. Each day is important. There are 24 ​h in a day. I may have laughed more (since I had MBC) (Omitted). I have changed. I feel like I've normally been living without thinking about anything. It's not that I'm not going to face death. I was facing death anyway, but the angle changed a little now, a little bit with people. It's like they finally realized what kind of person I am. I have come to think of it that way (Participant F).

Every day is so chaotic, but I can live with my daughter at home, and I can see my daughter's growth. That's what makes me happy. I'm happy to be able to spend time with people, and I think that time is irreplaceable (Participant J).

(7) Encouragement from strong supporters

This category involved receiving emotional, informational, and instrumental support from family members, patients with the same disease, and medical personnel when continuing painful cancer pharmacotherapy after diagnosis.

The words of my attending nurse have always comforted me. She said that because I did my best and received cancer pharmacotherapy eight years ago, the cancer has not recurred since then. So, it was meaningful for me to have worked hard and received cancer pharmacotherapy eight years ago. (Omitted) I am so thankful to her. I am so grateful (Participant B).

My husband and my younger sister have done quite a lot of research for me. And she has accompanied me for the hospital. I had the support of my family. Maybe my husband and my sister have a big presence (Participant I).

## Discussion

### Characteristics of the process from MBC diagnosis to the start of cancer pharmacotherapy

It has been reported that cancer metastasis makes the patient lose the possibility of cure,^23^ triggers anxiety about hastened death, and increases existential anxiety about the meaning and purpose of life and death.^24^ The other stresses experienced by patients with MBC include fear of dying, fear of disease progression and debilitation, and loss of the future.^5^ Therefore, the participants may have experienced the “threat of death” from having to confront mortality due to their breast cancer metastasizing.

Afterward, the participants were in “conflict with painful cancer pharmacotherapy” because there was “glimmer of hope provided by cancer pharmacotherapy,” but they felt “a sense of resistance to cancer pharmacotherapy based on past experiences.” Thirteen participants had received chemotherapy during their initial treatment for breast cancer. Such patients experienced physical and emotional burden and were reportedly traumatized by their side effects.^25^ Therefore, when the participants were diagnosed with MBC by a doctor and informed about the course of treatment, they were possibly reminded of their past traumas. This may have contributed to their resistance to cancer pharmacotherapy. However, the participants understood that rejecting the cancer pharmacotherapy would result in their death, possibly triggering an internal conflict. In the face of their conflict, the participants reconfigured their lives with the “encouragement from strong supporters.” They also proceeded with cancer pharmacotherapy upon reconciling.

Therefore, it is important for nurses to attend medical examinations at the time of MBC diagnosis to assess the patient's emotional state and initiate psychological support from an early time. Through dialogue, it is necessary to assist the patient in making sense of their past treatments, while helping them manage any side effects and improve their living environment so that they can positively engage in their treatment.

### Characteristics of the process from initiation of cancer pharmacotherapy to psychological adjustment

The participants experienced a “struggle to internalize MBC,” in which they repeatedly felt “aversion to not being able to escape breast cancer” and “hope to be kept alive by cancer drug treatment” when the cancer drug treatment began. Treatment for MBC aims to prolong life and relieve symptoms and lasts until therapeutic effects are seen. Since patients are said to experience difficulties in living with the uncertainty of MBC.^26^ The participants were believed to have recognized the fact that they must live in an uncertain state without being free of their breast cancer and cancer pharmacotherapy for the rest of their lives and felt “aversion to not being able to escape breast cancer.” However, the participants came to harbor a “hope to be kept alive by cancer pharmacotherapy” by virtue of their experience. One of the driving forces behind continuation of chemotherapy of patients with cancer is the anticipation of the effects of chemotherapy, and it is said that this anticipation encourages patients to fight cancer and live their lives to the fullest.^27^ Therefore, feeling the effects of the treatment is an important factor for patients to regain their sense of self.

Participants went from experiencing the “struggle to internalize MBC” to being able to “efforts to internalize MBC” with the “encouragement from strong supporters.” Social support has been shown to reduce anxiety and depression in patients with breast cancer, improve QOL,^8^^,^^28^ and even lead to adjustment to a breast cancer diagnosis.^29^

Therefore, an “encouragement from strong supporters” involves an important factor in the process of participants moving from “struggle to internalize MBC” to “efforts to internalize MBC.” It will be important for nurses to assess the social resources of patients with MBC and help them to utilize social support.

The participants made “efforts to internalize MBC,” leading to an “expansion of self-awareness.” After being diagnosed with MBC, the participants had negative emotions and conflicts, and they refused to accept the diagnosis. However, they were probably able to accept MBC gradually, through the “encouragement from strong supporters” and their own “shift to positive thinking,” as well as “attempt to live a normal life.” It has been reported that patients with MBC attempt to regain a sense of normalcy in their lives to “live well” despite their condition.^30^ In this way, the change in thinking to free oneself from obsessing over cancer or death and the effort to lead a normal life are important coping strategies for maintaining the psychological health of patients. Despite finding themselves in harsh circumstances, the participants looked at the big picture and realized that cancer had changed their values and outlook on life, leading to psychological growth. Posttraumatic growth (PTG) occurs in patients with cancer through self-disclosure and intentional ruminations, integrating traumatic events and creating some new meaning.^31^ In other words, the “expansion of self-awareness” in this study can be considered a state of PTG. Breast cancer survivors have been reported to experience PTG,^32^ and the fact that PTG was also observed in patients with MBC is a new finding in this study. Therefore, it will be important for nurses to believe that patients with MBC has the ability to grow despite their predicament and to provide systematic and ongoing support from the time of MBC diagnosis onward to help patients use effective coping strategies to achieve an expanded sense of self.

### Limitations

Study limitations include the small sample size and the inclusion of patients with MBC between the ages of 20 and 65. Future studies should increase the sample size and revalidate the study in patients with MBC over the age of 65. It is necessary to clarify the process of reaching psychological adjustment among patients with MBC who score 11 or higher on the HADS in the future. But in this study, potentially clinically useful results were obtained because M-GTA—an analysis method closely related to data—was used.

## Conclusions

Being diagnosed with MBC and subsequently receiving information about the treatment course forces patients to confront their mortality and experience an internal conflict over receiving cancer pharmacotherapy. However, with the support of family members and medical professionals, the patient decides to undergo the treatment. After starting treatment, the patient initially struggles to internalize their diagnosis. However, they begin to make efforts to internalize it with the support of family members and medical staff. Consequently, their self-awareness grows. Based on the results, a nursing intervention program should be developed that facilitates the psychological adjustment of patients.

## CRediT author statement

Author certifies that they have participated sufficiently in the work to take public responsibility for the content, including participation in the concept, design, analysis, writing, or revision of the manuscript. Furthermore, author certifies that this material or similar material has not been and will not be submitted to or published in any other publication before its appearance in the *Asia-Pacific Journal of Oncology Nursing*.
